# Supplementary material for: Anomalous size effect on yield strength enabled by compositional heterogeneity in high-entropy alloy nanoparticles
Source: Nat Commun. 2022 May 19;13:2789. doi: 10.1038/s41467-022-30524-z (PMC9120133; doi:10.1038/s41467-022-30524-z)
Supplement: Supplementary file 1 — Supplementary Information [file 41467_2022_30524_MOESM1_ESM.pdf]

SUPPLEMENTARY INFORMATION

Anomalous size effect on yield strength enabled by compositional heterogeneity in high-entropy alloy nanoparticles

Jingyuan Yan[1†], Sheng Yin[2], Mark Asta[2,3], Robert O. Ritchie[2,3], Jun Ding[4*] & Qian Yu[1*]

[1] Center of Electron Microscopy and State Key Laboratory of Silicon Materials, Department of Materials Science and Engineering, Zhejiang University, 310027, Hangzhou, China

[2] Materials Sciences Division, Lawrence Berkeley National Laboratory Berkeley, CA, 94720, USA

[3] Department of Materials Science and Engineering, University of California, Berkeley, CA, 94720, USA

[4] Center for Advancing Materials Performance from the Nanoscale (CAMP-Nano), State Key Laboratory for Mechanical Behavior of Materials, Xi'an Jiaotong University, Xi'an 710049, China

[†] Present Address: Institute of Engineering Innovation, The University of Tokyo, Tokyo 113-8656, Japan

*Correspondence to: dingsn@xjtu.edu.cn; yu_qian@zju.edu.cn.

Inventory of Supplementary Information

Supplementary Notes

Supplementary Note 1. Detailed discussion of the sample temperature during the TEM observation

Supplementary Figures

Supplementary Fig. 1 HADDF images of the FeCoNiCuPdIrPtAu nanoparticles.

Supplementary Fig. 2. MD simulation of the distribution of the stacking-fault energy (SFE) for the CrCoNi alloys with different sizes of stacking faults of L ~ 20 nm, 10 nm and 5 nm.

Supplementary Fig. 3. The simulated stress needed to move the dislocations in modeled CrCoNi, τ_{dis} *vs*. the stacking-fault energy γ_{SFE} in the random solid-solution sample and in samples annealed at 1350 K, 950 K and 650 K.

Supplementary Fig. 4. MD/MC simulation showing the difference in the value of L_{hetero} for the random solid solution and after annealing the CrCoNi simulation samples.

Supplementary Fig. 5. MD study of the relationship between $L_{critical}$ and extent of local heterogeneity.

Supplementary References

Supplementary Notes

Supplementary Note 1. Detailed discussion of the sample temperature during the TEM observation

The energy that the sample obtained during the experiment is mainly due to the energy loss of the electrons caused by collision and bremsstrahlung. According to the Bethe-Bloch equation[2],

$$Q = -\frac{d\bar{E}}{dx} = \frac{2\pi Z\rho(e^2/4\pi\varepsilon_0)^2}{mv^2}\left\{\ln\left[\frac{E(E+mc^2)^2\beta^2}{2I_e^2mc^2}\right] + (1-\beta^2) - \left(2-\sqrt{1-\beta^2}-1+\beta^2\right)\ln 2 + \frac{1}{8}(1-\sqrt{1-\beta^2})^2\right\} \quad (1),$$

where Z is the atomic number of the target element, and ρ is the atomic density. Here $\beta = \frac{v}{c}$, with c equal to the speed of light and v is the electron velocity. ε_0 is the vacuum permittivity, and e and m the electron charge and rest mass. E is the electron energy, and I_e is the average excitation energy for electrons in the target. Adopting the average value of the alloying element, the energy loss per length of the 200 kV-accelerated electrons in our HEA particles, namely the stopping power Q, is calculated to be 1.907 eV/nm. The e-beam energy is transferred to the sample at an energy density rate of

$$H = \frac{QJ}{e} = 3.43 \times 10^7 \text{ Wm}^{-3} \quad (2),$$

where J is the electron current density (1.8×10^{-2} Acm^{-2}) and e is the elementary charge (1.6×10^{-19} C).

Given above analysis, the energy transfer power P can be calculated from the volume V of the particle by:

$$P=HV \quad (3).$$

Note that the result of the particles with a diameter over 150 nm might be larger than the real situation since the decay distance of the electrons cannot reach such a dimension, which would result in the poor contrast in the middle of the particle in the TEM images (Fig.1). Here we take the extreme situation, assuming all particles (with diameter from 80 to 260 nm) are electron transparent and calculate the energy transfer. The results are listed in Supplementary Table 1.

Supplementary Table 1. The energy transfer power of particles with different sizes

Diameter (nm)	Energy transfer power P (10^{-11} W)
80	9.19
140	49.29
180	104.77
200	143.71
260	315.73

During the *in situ* experiment, the particle is in contact with the silicon wedge and diamond tip, which are of infinite size compared to the particles. The thermal conductivities of the silicon and diamond are 149 W·m^{-1}K^{-1} and 3320 W·m^{-1}K^{-1}, respectively, and thus can be deemed as ideal cold sinks. Therefore, the heat accumulation in the sample is determined by the ability of thermal transportation of the interface (interface thermal conductance), which is widely discussed, ranging from <0.01 MW·m^{-2}K^{-2} to 1000 MW·m^{-2}K^{-1} depending on the contacting situation[3-5]. In our study, the asperity of the nanoscale contacting interface would be easily eliminated with the application of high pressure because of plastic fit[1], which yields a high interface thermal conductivity close to that of the epitaxy or deposition. In fact, our Supplementary Movie 2 shows that considerable force is needed to pull the sample and probe apart after compression, which would overcome the strong adhesion applied at the interface. There have been some reports on the contact thermal conductance in such situations, including that of the Pt-Rh probe of scanning thermal microscope and various sample surfaces[6], adhered microcantilevers on the substrate[7], as well as the contact between wafer-like Al/Si solid surfaces under ~10 MPa pressure[8]. Values of the thermal conductivity G in these cases are all reported to be larger than 1 MW·m^{-2}K^{-1}. We thus take G=1 MW·m^{-2}K^{-1} for following analysis, considering the intimate contact between the particle and the silicon wedge and diamond tip[1]. This yields a temperature difference at the interface of:

$$\Delta T = \frac{HV}{GA} \quad (4),$$

where *V* is the sample volume and *A* is the total contact area, which is measured from the frames extracted from the videos. Taking the contacting area measured from the video, values of the temperature difference ΔT between the contact interface during the deformation process are listed in Supplementary Table 2. The largest temperature difference come up at the beginning of the compression, where the contacting area is smallest. During the compression, the contacting area increase rapidly and temperature differences at the interface further decrease. In this research, the maximum of ΔT is ~20.11 K (note the real temperature difference is even smaller because of the limited electron passing distance and a smaller energy transfer power *P* discussed previously), which is negligible compared to its melting temperature (over 1000 K).

Supplementary Table 2. The temperature difference ΔT during deformation process (unit: K)

Particle diameter \ Strain	0%	10%	20%	30%
80 nm	14.65	0.09	0.065	0.037
140 nm	19.62	0.13	0.064	0.049
180 nm	18.53	0.018	0.067	0.034
200 nm	14.3	0.019	0.085	0.028
260 nm	20.11	0.021	0.096	0.042

Based on above discussion, we know that the heat accumulated at the interface is negligible. However, the temperature inside the sample is not uniform and the temperature at the center of the sample is slightly higher than that of the interface due to the rather low thermal conductivity of the HEA (k~30 $\mathrm{Wm^{-1}K^{-1}}$) [9,10]. At equilibrium (which is evident from the minimal change in the level of the compression stress which would otherwise decrease if heat continuously accumulate inside the particle[1]), the temperature field inside the sample satisfies

$$k \nabla_{\vec{r}}^{2} T = -H \quad (5),$$

while at the hottest point (center of the particle in this case) the temperature gradient is zero. Then we have the temperature gradient $\nabla_{\vec{r}}^{2} T = -1.14 \times 10^{10}\ \mathrm{K{\cdot}m^{-2}}$ and the

temperature difference can be estimated with the sample dimension, to be small as 10^{-3} K, which means that during the compression process, the whole sample is as cold as the substrate and probe tip and the heat accumulation is negligible.

According to previous discussion, the sample temperature before the compression is slightly higher (about 10~20 K) than the environment; during the compression process the sample temperatures is kept same with the environment (room temperature).

Supplementary Figures

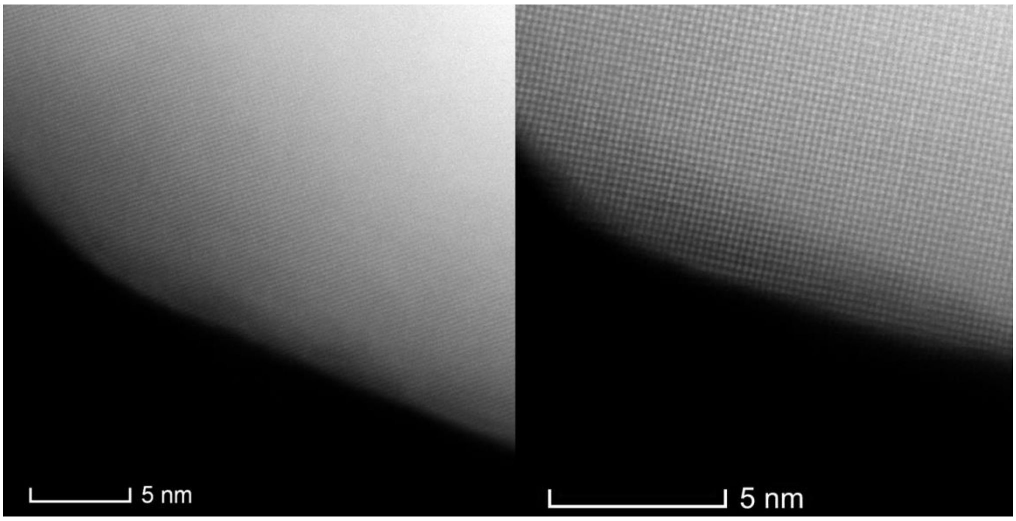

Supplementary Fig. 1. HADDF images of the FeCoNiCuPdIrPtAu nanoparticles.

It can be seen that there is no obvious element aggregation at the surface.

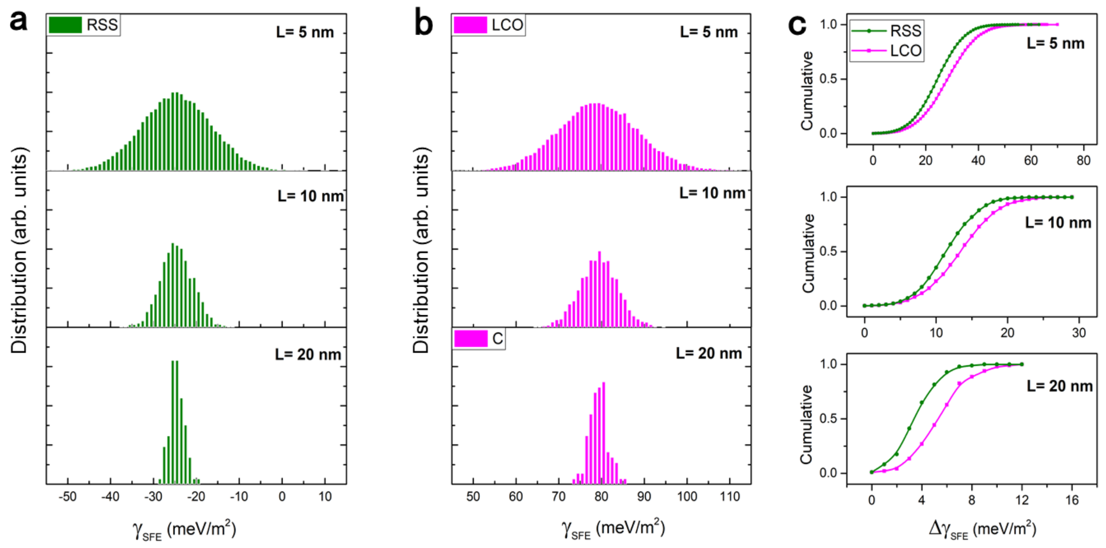

Supplementary Fig. 2. MD simulation of the distribution of the stacking-fault energy (SFE) for the CrCoNi alloys with different sizes of stacking faults of *L* ~ 20 nm, 10 nm and 5 nm. a, random solid-solution (RSS) configurations. **b,** Local chemical ordering (LCO) configurations annealed at 600 K. **c,** Cumulative ratio of the accrued SFE (defined as the difference between each SFE and the minimum one) for RSS and LCO configurations with a variety of *L* values. The considered stacking faults are considered to be square in shape with a width of *L*.

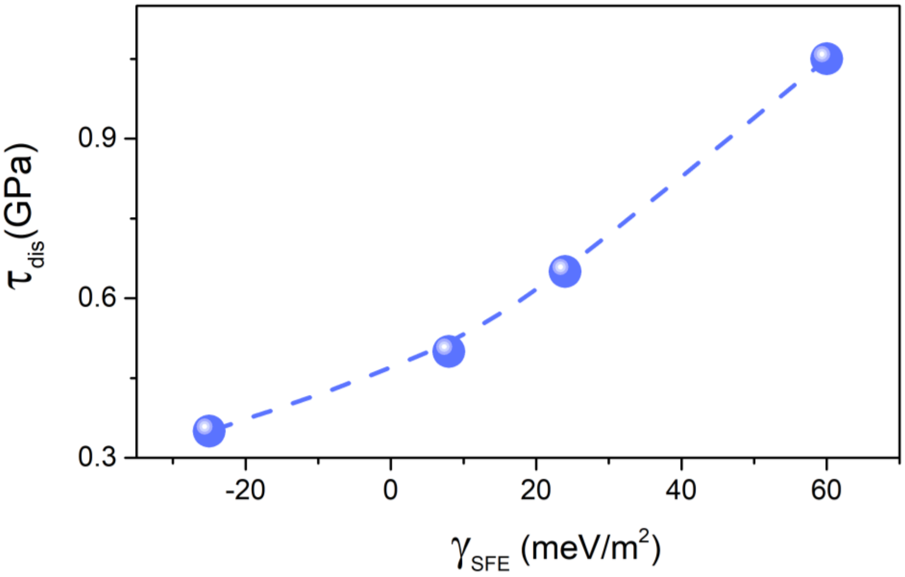

Supplementary Fig. 3. The simulated stress needed to move the dislocations in modeled CrCoNi, τ_{dis} *vs*. the stacking-fault energy γ_{SFE} in the random solid-solution sample and in samples annealed at 1350 K, 950 K and 650 K. τ_{dis} was determined by the stress to start moving the existing dislocation under simple shear at 300 K with a constant shear strain rate of 10^7 s^{-1}. (The SFE data and the τ_{dis} were respectively extracted from **Fig. 2f** and **Fig. 5c** in Ref. 17).

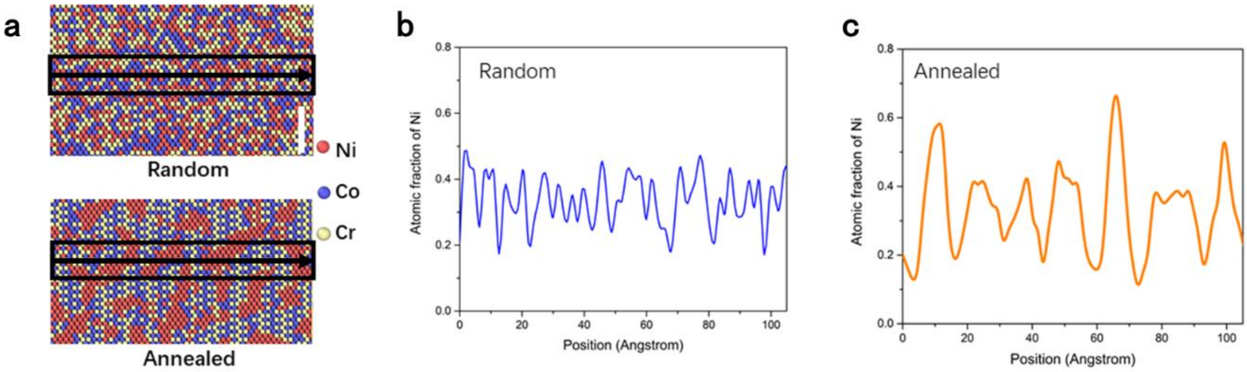

Supplementary Fig. 4. MD/MC simulation showing the difference in the value of L_{hetero} for the random solid solution and after annealing the CrCoNi simulation samples. a, Schematic description of the mapping of the atomic fraction of nickel along a random line in both the random solid-solution and annealed CrCoNi alloy samples (only the atoms within the radius of 1 nm from that line are counted, *i.e.*, the width of the line is 1 nm); (b) and (c) are the line profiles of the atomic fraction of nickel in the random solid solution and annealed (with LCO) CrCoNi alloy samples, respectively. L_{hetero} is determined as the averaged distance of the neighboring peaks in the line profile.

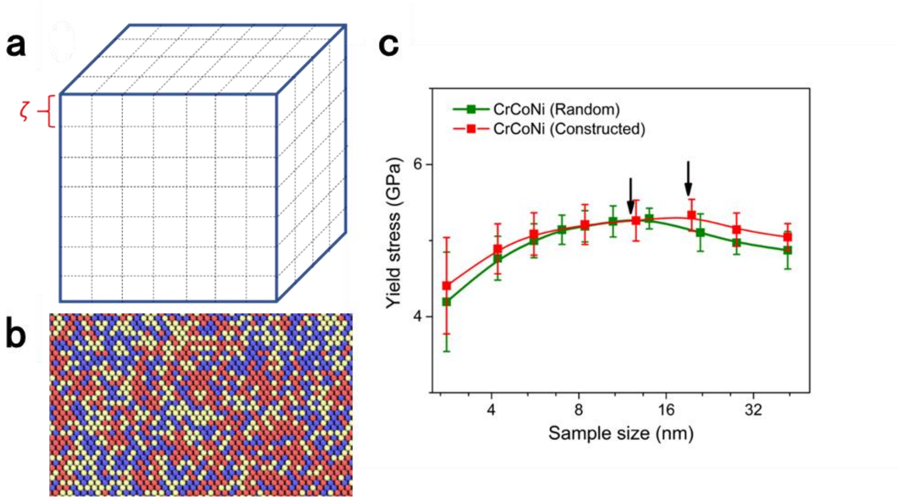

Supplementary Fig. 5. MD study of the relationship between $L_{critical}$ and extent of local heterogeneity. a, Schematic description of the artificially constructed CrCoNi alloy sample, which was divided into an array of cells with the size of each cell was set to ζ ~ 1 nm and the concentration of each cell was $(CrCo)_{1-x}Ni_x$, where *x* was randomly assigned between 0 and 2/3. **b,** An example of atomic configuration in {110} plane. **c,** The predicted yield stress *vs*. sample size for the nanowires of the CrCoNi alloy with random solid-solution atomic configurations versus artificially constructed configurations that have a larger correlation width for compositional heterogeneity (the error bars show the deviation of the results around the averaged values).

Supplementary References

1 Zheng, K. *et al.* Electron-beam-assisted superplastic shaping of nanoscale amorphous silica. *Nature Communications* **1**, 24 (2010).

2 Jencic, I., Bench, M., Robertson, I. & Kirk, M. Electron-beam-induced crystallization of isolated amorphous regions in Si, Ge, GaP, and GaAs. *Journal of Applied Physics* **78**, 974-982 (1995).

3 Lyeo, H.-K. & Cahill, D. G. Thermal conductance of interfaces between highly dissimilar materials. *Physical Review B* **73**, 144301 (2006).

4 Fletcher, L. Recent developments in contact conductance heat transfer. (1988).

5 Aikawa, T. & Winer, W. O. Thermal contact conductance across Si_3N_4—Si_3N_4 contact. *Wear* **177**, 25-32 (1994).

6 Lefèvre, S. & Volz, S. 3 ω-scanning thermal microscope. *Review of scientific instruments* **76**, 033701 (2005).

7 Huxtable, S. T., Cahill, D. G. & Phinney, L. M. Thermal contact conductance of adhered microcantilevers. *Journal of applied physics* **95**, 2102-2108 (2004).

8 Ohsone, Y., Wu, G., Dryden, J., Zok, F. & Majumdar, A. Optical measurement of thermal contact conductance between wafer-like thin solid samples. (1999).

9 Caro, M., Béland, L. K., Samolyuk, G. D., Stoller, R. E. & Caro, A. Lattice thermal conductivity of multi-component alloys. *Journal of Alloys and Compounds* **648**, 408-413 (2015).

10 Kush, L., Srivastava, S., Jaiswal, Y. & Srivastava, Y. Thermoelectric behaviour with high lattice thermal conductivity of Nickel base $Ni_2CuCrFeAl_x$ (x= 0.5, 1.0, 1.5 and 2.5) high entropy alloys. *Materials Research Express* **7**, 035704 (2020).

11 Warren, O. L., Downs, S. A. & Wyrobek, T. J. Challenges and interesting observations associated with feedback-controlled nanoindentation. *Zeitschrift für Metallkunde* **95**, 287-296 (2004).

12 Kumar, N. *et al.* High strain-rate compressive deformation behavior of the $Al_{0.1}CrFeCoNi$ high entropy alloy. *Materials & Design* **86**, 598-602 (2015).
