# Supplementary material for: Anomalous size effect on yield strength enabled by compositional heterogeneity in high-entropy alloy nanoparticles
Source: Nat Commun. 2022 May 19;13:2789. doi: 10.1038/s41467-022-30524-z (PMC9120133; doi:10.1038/s41467-022-30524-z)
Supplement: Supplementary file 3 — Description of Additional Supplementary Files [file 41467_2022_30524_MOESM3_ESM.pdf]

Description of Additional Supplementary Files

File Name: Supplementary Movie 1
Description: The deformation process of a 200 nm-sized particle. The deformation of the 200 nmsized particle is dominated by continuous dislocation movement. The particle was compressed into perfect “pie shape” with expansion in maximum width of over 30%.

File Name: Supplementary Movie 2
Description: The deformation process of a 180 nm-sized particle. Amounts of dislocation movements can be observed in the deformation process of 180 nm-sized particle while the whole process remained stable. The pulling process at the end shows the particle is in good contact with the wedge and probe.

File Name: Supplementary Movie 3
Description: The deformation process of a 140 nm-sized particle. Plastic instability was in evidence. Instable deformation with shear localization came up in the 140 nm-sized particle.

File Name: Supplementary Movie 4
Description: The deformation process of an 80 nm-sized particle. Fast dislocation movement and dislocation avalanche dominate the deformation process of the 80 nm-sized particle, resulting in catastrophic stress drop in the stress-strain curve.

File Name: Supplementary Movie 5
Description: The deformation process of a particle with twins. The deformation of this particle is initiated with dislocation nucleation in a single twin thus could be deemed as the yield process of a smaller particle.
